# Enhancing in-hospital mortality prediction in older patients with sepsis: the role of frailty indices and multidrug-resistance status in non-ICU wards—a proof-of-concept study

**DOI:** 10.1007/s40520-025-02955-3

**Published:** 2025-02-22

**Authors:** Chukwuma Okoye, Andrea Piazzoli, Maria Cristina Ferrara, Alberto Finazzi, Alice Margherita Ornago, Elena Pinardi, Beatrice Tonus, Paolo Mazzola, Andrea Ticinesi, Giuseppe Bellelli

**Affiliations:** 1 School of Medicine and Surgery, University of Milano-Bicocca, Milan, Italy; 2 Acute Geriatric Unit, IRCCS Foundation San Gerardo dei Tintori, Via Pergolesi, Monza, 33 - 20900 Italy; 3 Aging Research Center, Department of Neurobiology, Care Sciences and Society, Karolinska Institutet and Stockholm University, Stockholm, Sweden; 4 Internal Medicine Department, M.O.A Locatelli Hospital, ASST-Bergamo Est, Piario, Italy; 5 Department of Medicine and Surgery, University of Parma, Parma, Italy; 6 Department of Care Continuity and Multicomplexity, Parma University-Hospital, Via Antonio Gramsci 14, Parma, 43126 Italy

**Keywords:** Mortality, Sepsis, Multidrug resistance, Hospital, Frailty, Prediction

## Abstract

**Background:**

Prognostic stratification in older patients with sepsis is challenging due to frailty and the role of multidrug-resistant (MDR) infections.

**Aims:**

To test the predictive accuracy of different frailty measures, blood routine tests and MDR infection status for in-hospital mortality among older patients with sepsis.

**Methods:**

Consecutive patients aged ≥ 65 years with qSOFA ≥ 2 and positive cultures admitted to a tertiary care hospital were enrolled. Frailty was assessed using the Clinical Frailty Scale (CFS), the Primary Care–Frailty Index (PC-FI), and a 50-item FI. A base logistic regression model including age, sex, WBC count, platelets, creatinine, hs-CRP, and lactate predicted mortality. Frailty indices and MDR status were sequentially added, and model performance was compared using the area under the Receiver Operating Characteristics (AUROC). A nomogram was developed to visualize mortality probabilities.

**Results:**

Among 93 patients (median age 80, IQR [72–84] years, 63.4% males), in-hospital mortality was 16.1%. Deceased patients were frailer and had a higher number of comorbidities. By logistic multivariable regression, the base model achieved an AUROC of 0.771 for predicting in-hospital mortality. Adding frailty indices improved model performance to 0.800 (PC-FI), 0.817 (CFS), and 0.823 (FI). Incorporating MDR status further increased AUROC to 0.890 (PC-FI + MDR), 0.907 (CFS + MDR), and 0.922 (FI + MDR), outperforming the base model (*p* < 0.05 for all).

**Conclusions:**

Incorporating frailty indices and MDR status of culture isolates into traditional prognostic parameters improves mortality prediction in older patients admitted with sepsis, enabling more accurate risk stratification and personalized treatment strategies.

**Supplementary information:**

The online version contains supplementary material available at 10.1007/s40520-025-02955-3.

## Background

Sepsis, a severe life-threatening condition characterized by systemic organ dysfunction resulting from dysregulated host response to infection, is a leading cause of morbidity and mortality globally [1]. The burden of sepsis is especially high in older adults, who are most vulnerable to infections and severe complications. According to recent data [2], as compared with middle-aged and younger patients, older patients with sepsis face higher rates of septic shock (36% vs. 25% and 12%, respectively), 30-day mortality (17% vs. 6% and 4%), and chronic critical illness (42% vs. 34% and 22%).

Frailty is common among older adults and represents a state of increased vulnerability to stressors, reflecting multisystem physiological changes associated with aging and linked to an increased risk of negative outcomes [3]. Among older individuals with sepsis, frailty exacerbates the clinical course by further increasing the risk of negative health events [4]. While frailty measures have demonstrated prognostic value in Intensive Care Unit (ICU) settings for older individuals with sepsis and other critical illnesses [5–8], few studies have examined this relationship outside the ICU. Recent findings suggest that combining frailty measures with the Sequential Organ Failure Assessment (SOFA) score could enhance mortality prediction in geriatric wards [9, 10]; however, the generalizability of these findings in other hospital settings remains unexplored.

The growing prevalence of multidrug-resistant (MDR) infections is significantly complicating the management of sepsis in frail older adults, particularly those with culture-confirmed infections [11]. The rise of MDR pathogens in this population is associated with restricted antibiotic options, prolonged hospitalizations, and increased mortality [11]. Frequent exposure to healthcare settings, long-term care facilities, and invasive devices fosters a high risk of MDR colonization in older adults [12]. Given the impact of MDR on sepsis outcomes, integrating MDR status into predictive models alongside frailty indices could provide healthcare providers with more accurate and actionable insights to guide patient care.

This proof-of-concept study aims to assess the incremental prognostic value of incorporating various frailty indices and MDR status into traditional sepsis severity scores, with the objective of enhancing in-hospital mortality prediction among older patients hospitalized with sepsis and positive bacterial cultures in non-ICU acute care settings. Secondary aim was to develop a nomogram-like visual tool based on the significant determinants identified in the predictive models to stratify mortality risk and aid in clinical decision-making for older patients with sepsis.

## Methods

### Study design and population

This prospective, observational, longitudinal study was conducted at the Scientific Institute for Research, Hospitalization, and Healthcare (IRCCS) San Gerardo dei Tintori from November 1, 2022, to June 30, 2023. All patients who underwent infectious disease consultation for suspected sepsis during their stay in non-ICU acute wards were screened. Patients aged 65 years or older with confirmed diagnosis of sepsis and positive bacterial cultures on blood, urine, or other biological materials were included in this study. All patients underwent a Comprehensive Geriatric Assessment (CGA), performed by a well-trained resident in Geriatric Medicine during the infectious disease consultation. The CGA is a multidimensional evaluation of older adults, assessing medical conditions, functional status, comorbidities, nutrition, cognition, and frailty. It aids in risk stratification and individualized care planning, making it particularly relevant in geriatric sepsis management [13, 14].

Exclusion criteria included suspected sepsis with negative cultures, transfer to or from the ICU or a diagnosis of SARS-CoV-2 infection during hospitalization. The study conforms to the principles outlined in the Declaration of Helsinki. The protocol for this work was approved by the Regional IRB (protocol number 001421) on April 1st, 2022.

### Sepsis diagnosis and germs identification

Suspected sepsis was diagnosed according to the Sepsis-3 criteria (2016) by an infectious disease specialist during the consultation. Diagnosis was based on a quick Sequential Organ Failure Assessment (qSOFA) score of 2 or more points [15]. Patients were included in the study at the time of infectious disease consultation, which was conducted within the first 48 h of admission for nearly all cases. For patients admitted with suspected sepsis, this consultation was initiated based on clinical criteria, including signs of infection and organ dysfunction.

Peripheral venous blood samples were collected throughout the hospitalization period, with urine and other biological samples obtained as clinically indicated. Only bacteriological samples collected at the time of clinical suspicion of sepsis were included for diagnostic purposes. Culture analysis confirmed sepsis by identifying the causative organism and determining antimicrobial resistance using antibiotic susceptibility testing. To minimize the risk of misclassification due to contamination or colonization, all positive culture results were verified by the infectious disease specialist, using clinical correlation with symptoms and sepsis biomarkers. MDR was defined as acquired nonsusceptibility to at least one agent in three or more antimicrobial categories [16]. If multiple cultures were performed, MDR status was defined by the presence of at least one positive culture for a multidrug-resistant organism. Furthermore, MDR status was defined as the presence of multidrug-resistant organisms, detected either in confirmed infection or in clinically significant colonization. Colonization was included when MDR organisms were identified without clinical signs or symptoms of active infection but were deemed relevant by the infectious disease specialist in the context of the patient’s overall condition. Risk factors for MDR were also assessed, including recent hospitalization and/or antibiotic therapy, indwelling urinary catheter, central venous catheter, heart valve or orthopedic prostheses, recent surgical interventions, and admission from high-risk environments [17].

### Covariates

A comprehensive set of variables was collected during the first clinical interview using a CGA. These included patient demographics such as age, sex, body mass index (BMI), and social status, Charlson Comorbidity Index (CCI) [18], and polypharmacotherapy (total number of daily medications equal or greater than 5).

Pre-admission functional status was assessed using the Activities of Daily Living (ADL) and the Instrumental Activities of Daily Living (IADL) [19, 20]. Nutritional status was evaluated using the Mini Nutritional Assessment-short form (MNA-sf) [21] and calf circumference measurement.

Routine blood exams were collected at the time of sepsis diagnosis, including hemoglobin (Hb), white blood cells (WBC) and platelet counts, serum creatinine, high-sensitivity C-reactive protein (hs-CRP), procalcitonin (PCT), serum albumin, lactate. Additionally, vital sign data (e.g.) blood pressure, heart rate, peripheral saturation, respiratory rate), Glasgow Coma Scale (GCS), and other clinical instability scores (SOFA [22], NEWS2 [23] were collected.

Delirium was monitored throughout the hospital stay and categorized as prevalent if detected during the CGA on admission using the 4AT [24]. The 4AT is a rapid screening tool for delirium and cognitive impairment, widely used in acute care settings. It assesses four key domains: alertness, abbreviated mental test (AMT4), attention, and acute change or fluctuating course. A score of ≥ 4 suggests delirium, while 1–3 indicates possible cognitive impairment. It is efficient (≤ 2 min), does not require specialist training, and is validated for use in hospitalized older adults. Delirium was defined as incident if occurred during hospitalization, after the initial assessment, or persistent if identified during the CGA on admission and lasting for several days. Follow-up assessments for delirium were conducted in addition to CGA by reviewing medical records for keywords such as agitation, drowsiness, or confusion as documented by medical staff [25].

### Frailty assessment

Pre-admission frailty status was assessed using the Clinical Frailty Scale (CFS), the Primary Care Frailty Index (PC-FI) and a 50-item Frailty Index (FI). The 50-item FI was developed using variables derived from the CGA and laboratory data, following a standard procedure [26]. For each variable of the 50-item FI, a value of 0 was assigned in the absence of a deficit, and 1 in the presence of a deficit. For specific variables (e.g. incontinence, liver disease, diabetes mellitus, chronic kidney disease, and neoplasm), a value of 0 was assigned in the absence of a deficit, 0.5 in the case of mild-moderate deficit, and 1 in the case of severe deficit. Detailed information on the specific items and scoring criteria used to build the 50-item FI are provided in the supplementary materials (Supplementary Table 1). The threshold for defining frail patients were a score of 6 or higher on the CFS [27–29], a score of 0.14 on the PC-FI, and a score of 0.25 or higher on the 50-item FI.

### Outcome

The primary outcome was in-hospital mortality.

### Statistical analysis

Characteristics of the study population were reported using medians and interquartile ranges for continuous variables and absolute and relative frequencies for categorical variables. Group comparisons were conducted using the chi-square test for categorical variables and the Mann-Whitney U test for continuous variables. Univariable and multivariable logistic regression analyses were conducted to identify significant predictors of in-hospital mortality. The base model included age, sex, WBC count, platelet count, serum creatinine, hs-CRP, and lactate based on recent studies indicating those as factors associated with in-hospital mortality [30, 31]. The selection of variables for the multivariate analysis combined univariate analysis results with evidence from the literature to maximize the model’s predictive capacity. Variables such as platelet count, neutrophil count, and creatinine level were included despite limited statistical significance in our sample, as they are established predictors in larger studies. This approach mitigated the risk of excluding clinically relevant factors due to the small sample size, ensuring the model remained aligned with broader evidence. Models 1 A, 1B, and 1 C were constructed by adding the 50-item FI, the PC-FI, and the CFS, respectively. Models 2 (A, B, and C, respectively) further expanded Models 1 by including the MDR bacteria variable. Model performance was evaluated by assessing both discrimination and calibration. Discrimination was quantified using the area under the Receiving Operator Characteristics curve (AUROC). An AUROC of 0.5 indicates no discriminative ability, while an AUROC of 1 indicates perfect discrimination. Comparisons of AUROC between models were conducted using the DeLong test. Calibration was assessed using calibration plots and the Hosmer-Lemeshow goodness-of-fit test. Model calibration was further validated using bootstrap resampling techniques. Finally, a nomogram was created to visualize predicted mortality probabilities across CFS scores (2–8), categorized lactate levels (< 2 vs. ≥2), and culture status (non-MDR vs. MDR). Using multivariable logistic regression, predicted probabilities and 95% confidence intervals were computed and displayed in a heatmap, illustrating the impact of frailty, lactate, and antimicrobial resistance on mortality risk. The level of statistical significance was set at *p* < 0.05. All analyses were performed using JASP software version 0.17.3.0 and R software version 4.3.0 (The R Foundation for Statistical Computing, Vienna, Austria).

## Results

### Baseline characteristics

The baseline characteristics of the study population are summarized in Table 1. Initially, 125 patients diagnosed with sepsis by an infectious disease specialist during consultation were identified by geriatric specialty trainees. Of these, 32 patients were subsequently excluded based on predefined criteria: 8 were transferred to or from the ICU, 5 had a diagnosis of SARS-CoV-2 infection during hospitalization, 11 had a qSOFA score < 2, and 8 had negative microbiologic culture results. The final study cohort thus comprised 93 patients (mean age 80 years, 37.6% females). Hospital-acquired sepsis was identified in three cases where symptoms and diagnostic criteria developed after admission. The median length of stay was 19 days (IQR: 12–27 days). The primary infections sources included urinary (20 cases), respiratory (7 cases), cardiac (3 cases), cutaneous (3 cases), and abdominal (8 cases). Forty-three cultures were positive for MDR organisms, with 25 detected in blood cultures, 17 in urine cultures, and 9 from other infection sites.

**Table 1:** Baseline characteristics of the study population

Variables	Overall (*n* = 93)	Alive (*n* = 78)	Dead (*n* = 15)	*p*-value
**Demographics**				
Age, years	80 (72–84)	79.5 (72–83)	84 (77.5–87)	0.06
Sex, male (n, %)	59 (63.4)	48 (61.5)	11 (73.3)	0.38
**Health status**				
ADL	5 (1–6)	5 (1.25–6)	1 (1.0–3.5)	0.02
IADL	2 (0–5)	3 (1–5)	0 (0–1.5)	0.002
Polypharmacotherapy (n, %)	67 (72)	53 (67.9)	14 (93.3)	0.13
CCI	6 (5–8)	5.5 (4.2–8)	8 (7–9)	0.009
Dementia (n,%)	31 (33.3)	23 (29.5)	8 (53.3)	0.07
MNA sf	11 (8–12)	11 (8–13)	8 (7.5–11)	0.10
Calf circumference	32 (29–35)	32 (30–36)	29 (27.5–31)	0.01
50-item FI	0.26 (0.16–0.37)	0.23 (0.15–0.34)	0.36 (0.29–0.41)	0.004
50-item FI ≥ 0.25 (n, %)	48 (51.6)	35 (44.87)	13 (86.67)	0.003
PC-FI	0.16 (0.08–0.24)	0.16 (0.08–0.24)	0.24 (0.2–0.28)	0.006
PC-FI ≥ 0.15 (n,%)	58 (62.4)	44 (65.4)	14 (93.3)	0.007
CFS	6 (5–7)	6 (5–7)	7 (6–7)	0.028
CFS 6–9 (n, %)	58 (62.4)	44 (56.4)	14 (93.3)	0.007
Delirium (n, %)	60 (64.6)	46 (58.9)	14 (93.3)	0.011
**Laboratory serum levels**				
Hemoglobin, g/dL	11.3 (9.7–13.2)	11.6 (9.7–13.2)	11 (9.3–13.4)	0.78
creatinine, mg/dL	1.4 (0.9–2.1)	1.3 (1–1.8)	1.6 (0.9–2.4)	0.57
Albumin, g/dL	2.9 (2.5–3.3)	2.9 (2.6–3.3)	3 (2.4–3.2)	0.79
WBC, x10^3/uL	12.47 (7.63–17.48)	12.0 (7.70–17.4)	16.6 (8.20–20.5)	0.42
Lactate, mmol/L	1.9 (1.3–2.5)	1.7 (1.2–2.5)	2.3 (1.6–3.1)	0.08
Hs-CRP, mg/dL	11.8 (6.5–19.0)	13.1 (7.0–19.3)	7.6 (4.6–13.8)	0.13
Procalcitonin, ng/mL	1.9 (0.6–14.6)	1.9 (0.6–15.7)	1.8 (0.5–9.2)	0.79
SOFA score (median)	5 (2–8)	4 (1–3)	6 (4–8)	0.063
Presence of at least one positive culture for MDR bacteria	42 (45.2)	28 (36.0)	14 (93.3)	0.001

Continuous variables are expressed as mean and SD or median with IQR properly

Abbreviations: ADL = Activities of Daily Living, IADL = Instrumental Activities of Daily Living, CCI = Charlson Comorbidity Index, MNA sf = Mini Nutritional Assessment short form, FI = Frailty Index, PC-FI = Primary Care Frailty Index, CFS = Clinical Frailty Scale, WBC = White Blood Cell, Hs-CRP = High-sensitivity C-reactive protein, MDR = Multidrug resistant.

### Relation between frailty, MDR bacteria and in-hospital mortality

**Fig. 1:**
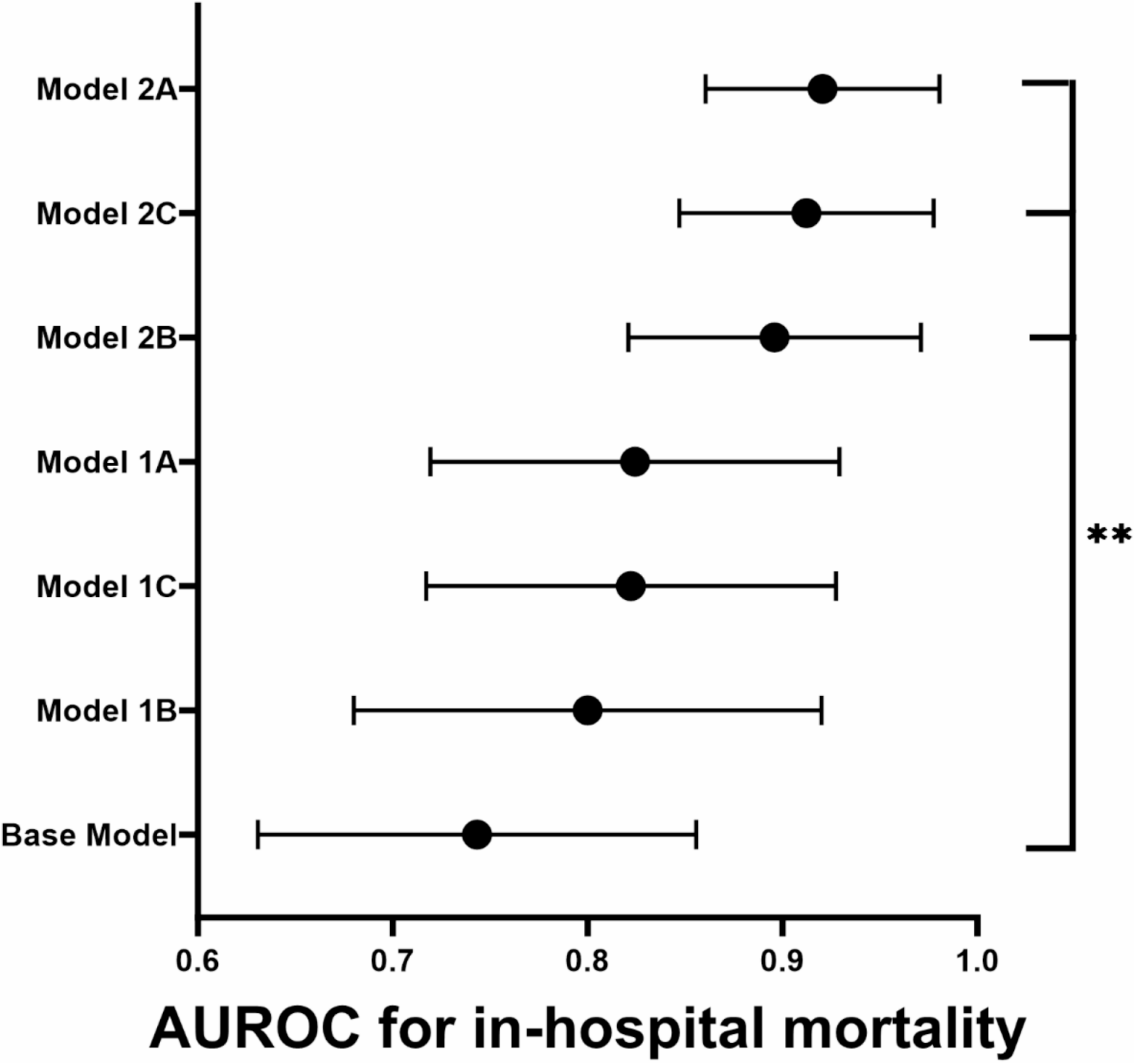
Area under the receiving operator characteristics curve (AUROC) for in-hospital mortality of multivariable logistic regression models

During the hospital stay, 15 patients (16.1%) died. No significant differences in admission parameters were observed between deceased patients and survivors (Supplementary Table 2). Results of the univariable regression analyses are presented in Supplemental Table 3. Multivariable logistic regression analysis showed that the base model—which included age, sex, WBC count, platelet count, serum creatinine, hs-CRP and lactate—achieved an AUROC of 0.771, indicating moderate ability to discriminate between survivors and non-survivors (Supplementary Table 4). Higher age (OR 1.10, 95% CI 1.02–1.21, *p* = 0.029) and lactate levels (OR 1.45, 95% CI 1.07–2.04, *p* = 0.020) were significantly associated with increased in-hospital mortality. Adding frailty measures to the base model significantly improved its predictive performance (Fig. 1). Model 1 A, which included the 50-item FI demonstrated an AUROC of 0.823, with the FI emerging as a strong predictor of in-hospital mortality (*p* = 0.018) (Supplementary Table 5). Model 1B, which incorporated PC-FI (Supplementary Table 6), achieved a AUROC of 0.800. Model 1 C, which included the CFS, showed an AUROC of 0.817. The CFS was also a significant predictor of in-hospital mortality (*p* = 0.044, Supplementary Table 7). In Model 2, adding positive cultures for MDR bacteria to Models 1 A, 1B, and 1 C further improved predictive performance (Fig. 1), reaching statistical significance at the DeLong test (*p* < 0.05 for each), compared to Base Model. Model 2 A (50-item FI + MDR) achieved an AUROC of 0.922, Model 2B (PC-FI + MDR had an AUROC of 0.890, and Model 2 C (CFS + MDR reached an AUROC of 0.907, indicating excellent predictive accuracy.

**Fig. 2:**
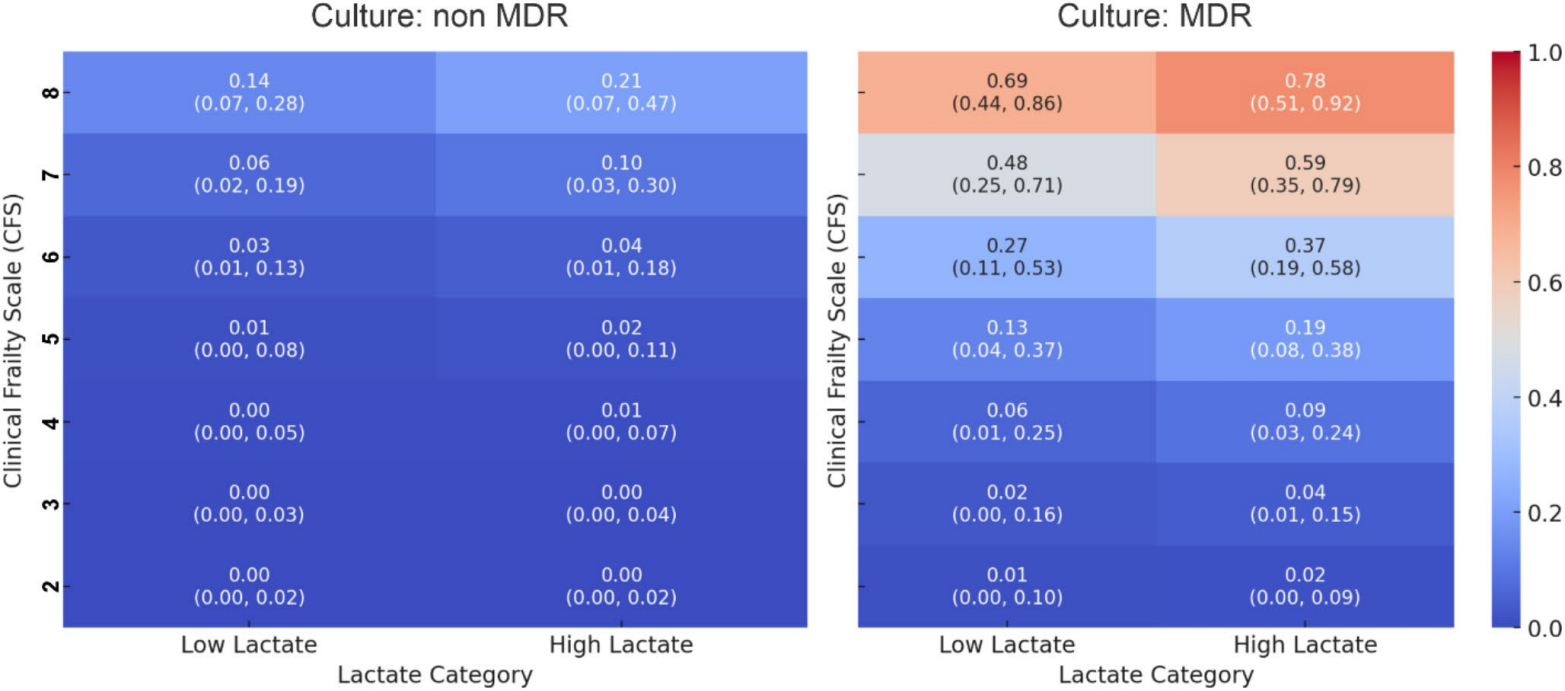
Nomogram-like plot with confidence intervals: predicted mortality probability by Clinical Frailty Scale, lactate category, and culture status

Finally, a nomogram was developed using Model 2 C, given its simplicity and the widespread use of the CFS in acute geriatric units (Fig. 2). The analysis indicated that higher predicted mortality was associated with CFS scores of 6 or greater, elevated lactate levels (≥ 2 mmol/L), and MDR bacterial status. Patients with a CFS score of 7 or 8, combined with high lactate and MDR, had the highest mortality risk. In contrast, lower CFS scores, normal lactate levels, and non-MDR status were associated with substantially lower predicted mortality.

## Discussion

This study demonstrated that integrating frailty assessments with MDR culture status significantly improves the prediction of in-hospital mortality in older patients with sepsis. The combination of the 50-item FI and positive MDR status showed the highest predictive accuracy, outperforming models based solely on demographic or laboratory markers. Moreover, the nomogram highlighted the particularly high risk of death in patients with severe frailty (CFS ≥ 7) and positive MDR cultures. This compounded risk highlights a critically vulnerable group of patients that requires prioritized and tailored care. Testing this combined predictive model in larger, multicenter cohorts is essential to confirm its robustness and generalizability, ensuring its reliability in guiding clinical decision-making across varied healthcare settings.

Various studies have shown that frailty is associated with adverse clinical outcomes and mortality among patients with sepsis [32–34]. A recent prospective cohort study, including 211 participants, demonstrated that frailty significantly impacts the one-year prognosis in older patients hospitalized for sepsis [35]. Another recent study, analyzing data from 240 patients consecutively admitted to an acute geriatric unit, showed that the combined use of the SOFA score with either the FI or the CFS improves the accuracy of predicting both short- and long-term mortality.

Consistent with prior research on sepsis outcomes, our models identified both age and elevated lactate levels as significant predictors of in-hospital mortality [9, 36]. However, the moderate predictive accuracy of this model suggests that traditional risk factors alone fail to fully capture the complex dynamics affecting short-term mortality in older patients with sepsis. Frailty, which arises from an accelerated accumulation of age-related health deficits, significantly enhances both susceptibility to infections and the severity of infection-related complications [11]. In our cohort, the addition of frailty assessment to the traditionally used variables significantly improved mortality prediction, with an AUROC increasing to 0.800 and over across all models. A nationwide propensity score-matched cohort study by Lee et al. showed that preexisting frailty doubled the risk of in-hospital mortality in a large sample of adults hospitalized with sepsis [32]. These findings highlight the importance of incorporating CGA into the current management of older patients with sepsis, to effectively evaluate the multifaceted and heterogeneous health profiles of older individuals.

Importantly, our analysis revealed no significant differences among the various tools employed to assess frailty, suggesting that what truly matters is assessing frailty itself, regardless of the specific tool employed. Notably, although the CFS score may be influenced by subjective judgment, we employed the Theou et al. [28] CFS standardized algorithm to enhance the reliability and consistency of assessments. The greater variability observed for the PC-FI in our analyses, compared to other frailty tools, may be attributable to its primary validation in community-dwelling populations.

It is noteworthy that, besides frailty, MDR in various cultures has also been independently associated with poor outcomes in sepsis [37]. A recent study showed that frail patients had similar rates of infection than non-frail patients but were more prone to have MDR microorganisms as causative pathogens [38]. However, the interaction between frailty, MDR and poor outcomes remains underexplored. In our analysis, incorporating data on MDR infections further enhanced the predictive performance of models, highlighting a compounded effect of frailty and MDR on mortality risk. Frail patients may have limited physiological reserve to withstand with the systemic stress of sepsis, which can lead to rapid deterioration during severe and MDR infections [39]. On the other side, MDR, with its potential to complicate treatment regimens and prolong hospital stays [38], may hinder patients’ ability to restore homeostasis and exacerbate frailty. This interplay may contribute to a vicious cycle that significantly impacts in-hospital mortality in older individuals.

The results of this study carry several important implications for clinical practice in older patients hospitalized with sepsis. Integrating frailty assessments with data on positive MDR cultures may significantly improve the accuracy of predicting in-hospital mortality. This combined approach enables better identification of high-risk patients compared to models that rely only on demographic data or common laboratory biomarkers. Incorporating frailty measures into routine evaluations provides a more comprehensive understanding of the patient, while including MDR status in predictive models emphasizes the critical role of addressing antibiotic resistance in clinical settings. With a more accurate prediction model, healthcare professionals can implement personalized and timely interventions, optimizing resources allocation and improving patient outcomes.

### Strengths and limitations

This study expands the current literature on sepsis and mortality in older adults by including older patients hospitalized in various medical wards. Additionally, it benefits from using cultures to identify MDR bacteria presence. Other strengths include its prospective design and the thorough implementation of a comprehensive geriatric assessment, conducted by a well-trained examiner, including frailty assessment. However, we also acknowledge some limitations that are worth mentioning. First, the small sample size and the single-center design may limit the generalization of our findings. Second, the study was limited to patients referred to infectious disease consultation services, which may have introduced a selection bias towards individuals with greater clinical complexity and higher likelihood of infections by MDR pathogens. Third, no adjustment was made for infection sites and antibiotic therapy. Furthermore, we acknowledge that the reliance on non-invasive sampling may have excluded certain pulmonary infections requiring invasive diagnostic procedures, particularly in frail patients.

## Conclusions

This study demonstrates that the integration of frailty assessment and MDR status of blood cultures to traditional prognostic parameters of sepsis may improve in-hospital mortality prediction in older patients admitted to various non-ICU acute settings. Thus, it represents a step forward in mortality risk prediction among older patients with sepsis, offering a novel approach through the combined assessment of frailty and antimicrobial resistance status. While promising, our findings could serve as a proof-of-concept, highlighting the need for a larger, multicenter study to validate and refine this approach, ensuring its applicability and robustness across varied clinical settings and patient populations.

## Electronic supplementary material

Below is the link to the electronic supplementary material.

Supplementary Material 1

## Data Availability

No datasets were generated or analysed during the current study.
