# Supplementary material for: Enhancing in-hospital mortality prediction in older patients with sepsis: the role of frailty indices and multidrug-resistance status in non-ICU wards—a proof-of-concept study
Source: Aging Clin Exp Res. 2025 Feb 22;37(1):45. doi: 10.1007/s40520-025-02955-3 (PMC11846750; doi:10.1007/s40520-025-02955-3)
Supplement: Supplementary file 1 — Supplementary Material 1 [file 40520_2025_2955_MOESM1_ESM.docx]

**Supplementary Table 1: 50-item Frailty Index**

|  | **Deficit** | **Cut-off** | | |
| --- | --- | --- | --- | --- |
|  |  | 0 | 0,5 | 1 |
| 1. | Dependency in Washing | Absent |  | Present |
| 2. | Dependency in Toileting | Absent |  | Present |
| 3. | Dependency in Feeding | Absent |  | Present |
| 4. | Dependency in Mobility | Absent |  | Present |
| 5. | Dependency in Dressing | Absent |  | Present |
| 6. | Incontinence | Absent | Single | Double |
| 7. | Dependency in Financial Management | Absent |  | Present |
| 8. | Dependency in Shopping | Absent |  | Present |
| 9. | Dependency in Telephone Use | Absent |  | Present |
| 10. | Dependency in Medication Management | Absent |  | Present |
| 11. | Dependency in Transport | Absent |  | Present |
| 12. | Reduced Nutritional Intake <3 Months | Absent |  | Present |
| 13. | Weight Loss > 3 kg < Months | Absent |  | Present |
| 14. | Calf Circumference < Sex-specific cut-off | Absent |  | Present |
| 15. | Albumin <3.4 g/dL | Absent |  | Present |
| 16. | Abnormal Hb | Absent |  | Present |
| 17. | Abnormal Creatinine | Absent |  | Present |
| 18. | Abnormal Sodium | Absent |  | Present |
| 19. | Abnormal Potassium | Absent |  | Present |
| 20. | Abnormal Aspartate Aminotransferase | Absent |  | Present |
| 21. | Abnormal Alanine Aminotransferase | Absent |  | Present |
| 22. | Ischemic Heart Disease | Absent |  | Present |
| 23. | Heart Failure | Absent |  | Present |
| 24. | Peripheral Arterial Disease | Absent |  | Present |
| 25. | Stroke/TIA | Absent |  | Present |
| 26. | Dementia | Absent |  | Present |
| 27. | COPD | Absent |  | Present |
| 28. | Connective Tissue Disease | Absent |  | Present |
| 29. | Peptic Ulcer | Absent |  | Present |
| 30. | Liver Disease | Absent | Mild - Moderate | Severe |
| 31. | Diabetes Mellitus | Absent | Not - complicated | Complicated |
| 32. | Hemiplegia | Absent |  | Present |
| 33. | Chronic Kidney Disease | Absent | Moderate | Severe |
| 34. | Cancer | Absent | Localized | Metastatic |
| 35. | Leukemia | Absent |  | Present |
| 36. | Lymphoma | Absent |  | Present |
| 37. | AIDS | Absent |  | Present |
| 38. | Arterial Hypertension | Absent |  | Present |
| 39. | Arrhythmias | Absent |  | Present |
| 40. | Valvular Heart Disease | Absent |  | Present |
| 41. | Thyroid Disorders | Absent |  | Present |
| 42. | Anemia | Absent |  | Present |
| 43. | Parkinson/Parkinsonism/Neuropathies | Absent |  | Present |
| 44. | Depression/Anxiety Disorders | Absent |  | Present |
| 45. | Dysphagia | Absent |  | Present |
| 46. | Visual Impairment | Absent |  | Present |
| 47. | Hearing Impairment | Absent |  | Present |
| 48. | Pressure Ulcers ≥ stage 2 | Absent |  | Present |
| 49. | Constipation | Absent |  | Present |
| 50. | Bed rest syndrome | Absent |  | Present |

Abbreviations: Hb = Hemoglobin, TIA = Transient Ischemic Attack, COPD = Chronic Obstructive Pulmonary Disease, AIDS = Acquired Immunodeficiency Syndrome.

| **Variables** | **OR** | **95% CI** | **p-value** |
| --- | --- | --- | --- |
| Sex |  |  |  |
| Female | — | — |  |
| male | 1.72 | 0.53, 6.65 | 0.4 |
| Age | 1.07 | 0.99, 1.16 | 0.082 |
| Basic activities of daily living | 0.73 | 0.55, 0.94 | 0.020 |
| Instrumental activities of daily living | 0.59 | 0.37, 0.83 | 0.007 |
| Charlson comorbidity index | 1.31 | 1.05, 1.68 | 0.021 |
| Calf circumference < 31 cm |  |  |  |
| no | — | — |  |
| yes | 3.78 | 1.21, 13.2 | 0.026 |
| Frailty index |  |  |  |
| < 0.25 | — | — |  |
| >= 0.25 | 7.99 | 2.03, 53.3 | 0.009 |
| Primary care Frailty Index |  |  |  |
| < 0.07 | — | — |  |
| >= 0.21 | 5.33 | 0.84, 105 | 0.13 |
| 0.07 - 0.14 | 0.00 | >0.9 |  |
| 0.14 - 0.21 | 3.65 | 0.54, 72.8 | 0.3 |
| Clinical Frailty Scale | 1.75 | 1.10, 3.19 | 0.036 |
| Serum creatinine | 1.05 | 0.64, 1.56 | 0.8 |
| Lactate | 1.28 | 0.99, 1.66 | 0.057 |
| Neutrophils | 1.04 | 0.96, 1.12 | 0.3 |
| Platelets | 1.00 | 1.00,1.00 | 0.6 |

**Supplementary Table 2: Univariable Logistic Regression Results for Mortality**

**Supplementary Table 3: Clinical parameters at hospital admission**

| **Variables** | **Overall**  **(n=93)** | **Alive**  **(n=78)** | **Dead**  **(n=15)** | **p-value** |
| --- | --- | --- | --- | --- |
| **Respiratory Rate** | **8 (8.6)** | **6 (7.7)** | **2 (8.4)** | 0.66 |
| 15-20 | 70 (75.3) | 60 (79.9) | 10 (66.6) |  |
| 21-29 | 15(16.1) | 12 (15.4) | 3 (20.0) |  |
| **≥ 30** |  |  |  |  |
| **Heart rate (beats per minute)** |  |  |  |  |
| 101-110 | 13 (13.9) | 13 (16.7) | 0 (0) | 0.16 |
| 111-129 | 8 (8.6) | 5 (6.4) | 3 (20.0) |  |
| 41-50 | 1 (1.1) | 1 (1.3) | 0 (0.0) |  |
| 51-100 | 66 (70.9) | 54 (69.2) | 12 (80) |  |
| ≥ 130 | 5 (5.4) | 5 (6.4) | 0 (0) |  |
| **Systolic Blood Pressure** |  |  |  | 0.64 |
| 101-199 | 28 (30.1) | 22 (28.2) | 6 (40.0) |  |
| 71-80 | 18 (19.3) | 16 (20.5) | 2 (13.3) |  |
| 81-100 | 44 (47.3) | 38 (48.7) | 6 (40) |  |
| ≤70 | 3 (3.2) | 2 (2.5) | 1 (6.4) |  |
| **Peripheral Saturimetry** |  |  |  | 0.67 |
| 91-94 % | 21 (22.6) | 18 (23.2) | 3 (20.0) |  |
| >90 % | 10 (10.7) | 8 (10.2) | 2 (13.3) |  |
| >94 % | 39 (41.9) | 34 (43.6) | 5 (33.3) |  |
| Venturi Mask | 21 (22.6) | 17 (21.8) | 4 (26.6) |  |
| Non-invasive ventilation | 2 (2.1) | 1 (1.3) | 1 (6.7) |  |

**Supplementary Table 4: Factors associated with in-hospital mortality. Multivariable regression analysis, base model.**

|  | OR*^1^* | 95% CI*^1^* | p-value |
| --- | --- | --- | --- |
| Age | 1.10 | 1.02, 1.21 | 0.03 |
| Male sex | 1.90 | 0.52, 8.28 | 0.35 |
| Serum Creatinine | 0.99 | 0.55, 1.62 | 0.98 |
| Serum Neutrophil | 1.05 | 0.95, 1.15 | 0.35 |
| Serum Platelet | 1.00 | 1.0, 1.00 | 0.87 |
| Serum Lactate serum | 1.45 | 1.07, 2.04 | 0.02 |
| Hs-CRP serum levels | 0.95 | 0.89, 1.01 | 0.18 |
| *^1^* OR = Odds Ratio, CI = Confidence Interval | | | |

Abbreviations: Hs-CRP = High-sensitivity C-reactive protein.

**Supplementary Table 5: Factors associated with in-hospital mortality. Multivariable regression analysis, base model plus 50-item Frailty Index (Model 1A)**

|  | OR*^1^* | 95% CI*^1^* | p-value |
| --- | --- | --- | --- |
| Age | 1.07 | 0.98, 1.18 | 0.2 |
| Male sex | 2.79 | 0.69, 14.3 | 0.2 |
| Serum Creatinine | 0.84 | 0.42, 1.44 | 0.6 |
| Serum Neutrophils | 1.06 | 0.95, 1.18 | 0.3 |
| Serum Platelets | 1.00 | 0.99, 1.00 | 0.8 |
| Serum Lactate | 1.47 | 1.08, 2.11 | 0.022 |
| Hs-CRP | 0.97 | 0.90, 1.03 | 0.5 |
| 50-item FI ≥0.25 | 8.45 | 1.76, 66.2 | 0.018 |
| *^1^* OR = Odds Ratio, CI = Confidence Interval | | | |

Abbreviations: Hs-CRP = High-sensitivity C-reactive protein, FI = Frailty Index.

**Supplementary Table 6: Factors associated with in-hospital mortality. Multivariable regression analysis, base model plus Primary Care Frailty Index (Model 1B)**

|  | OR*^1^* | 95% CI*^1^* | p-value |
| --- | --- | --- | --- |
| Age | 1.08 | 0.99, 1.19 | 0.11 |
| Male sex (ref) | 1.96 | 0.51, 9.34 | 0.45 |
| Serum Creatinine | 1.01 | 0.54, 1.67 | 0.96 |
| Serum Neutrophils | 1.05 | 0.94, 1.16 | 0.39 |
| Serum Platelets | 1.00 | 0.99, 1.00 | 0.92 |
| Serum Lactate | 1.46 | 1.08, 2.10 | 0.02 |
| Hs-CRP | 0.96 | 0.89, 1.02 | 0.24 |
| PC-FI > 0.14 | 0.36 | 0.10, 1.27 | 0.11 |
| *^1^* OR = Odds Ratio, CI = Confidence Interval | | | |

Abbreviations: Hs-CRP = High-sensitivity C-reactive protein, PC–FI = Primary Care Frailty Index

**Supplementary Table 7: Factors associated with in-hospital mortality. Multivariable regression analysis, base model plus Clinical Frailty Scale (Model 1C)**

|  | OR*^1^* | 95% CI*^1^* | p-value |
| --- | --- | --- | --- |
| Age | 1.07 | 0.99, 1.18 | 0.12 |
| Male sex (ref) | 2.57 | 0.69, 11.5 | 0.21 |
| Serum Creatinine | 0.92 | 0.49, 1.55 | 0.82 |
| Serum Neutrophils | 1.06 | 0.95, 1.18 | 0.34 |
| Serum Platelets | 1.00 | 0.99, 1.00 | 0.71 |
| Serum Lactate | 1.47 | 1.07, 2.10 | 0.021 |
| Hs-CRP | 0.96 | 0.89, 1.02 | 0.22 |
| CFS | 1.81 | 1.07, 3.49 | 0.044 |
| *^1^* OR = Odds Ratio, CI = Confidence Interval | | | |

Abbreviations: Hs-CRP = High-sensitivity C-reactive protein, CFS = Clinical Frailty Scale.

| **Variables** | **OR** | **95% CI** | **p-value** |
| --- | --- | --- | --- |
| **Sex** |  |  |  |
| **Female** | **—** | **—** |  |
| **male** | **1.72** | **0.53, 6.65** | **0.4** |
| **Age** | **1.07** | **0.99, 1.16** | **0.082** |
| **Basic activities of daily living** | **0.73** | **0.55, 0.94** | **0.020** |
| **Instrumental activities of daily living** | **0.59** | **0.37, 0.83** | **0.007** |
| **Charlson comorbidity index** | **1.31** | **1.05, 1.68** | **0.021** |
| **Calf circumference < 31 cm** |  |  |  |
| **no** | **—** | **—** |  |
| **yes** | **3.78** | **1.21, 13.2** | **0.026** |
| **Frailty index** |  |  |  |
| **< 0.25** | **—** | **—** |  |
| **>= 0.25** | **7.99** | **2.03, 53.3** | **0.009** |
| **Primary care Frailty Index** |  |  |  |
| **< 0.07** | **—** | **—** |  |
| **>= 0.21** | **5.33** | **0.84, 105** | **0.13** |
| **0.07 - 0.14** | **0.00** | **>0.9** |  |
| **0.14 - 0.21** | **3.65** | **0.54, 72.8** | **0.3** |
| **Clinical Frailty Scale** | **1.75** | **1.10, 3.19** | **0.036** |
| **Serum creatinine** | **1.05** | **0.64, 1.56** | **0.8** |
| **Lactate** | **1.28** | **0.99, 1.66** | **0.057** |
| **Neutrophils** | **1.04** | **0.96, 1.12** | **0.3** |
| **Platelets** | **1.00** | **1.00,1.00** | **0.6** |
